# Redefining Debt-to-Health, a triple-win health financing instrument in global health

**DOI:** 10.1186/s12992-024-01043-x

**Published:** 2024-05-06

**Authors:** Yunxuan Hu, Zhebin Wang, Shuduo Zhou, Jian Yang, Ying Chen, Yumeng Wang, Ming Xu

**Affiliations:** 1 Department of Global Health, Peking University School of Public Health, Haidian District, 38 Xue Yuan Road, Beijing, 100191 China; 2 Institute for Global Health and Development, Peking University, Beijing, China; 3 School of International and Public Affairs, Columbia University, New York, USA; 4 PATH Shanghai Representative Office, Shanghai, China

**Keywords:** Debt-to-Health, Review, Innovative health financing instrument, Global health

## Abstract

**Background:**

As a recognized win–win-win approach to international debt relief, Debt-to-Health(D2H)has successfully translated debt repayments into investments in health-related projects. Although D2H has experienced modifications and periodic suspension, it has been playing an increasingly important role in resource mobilization in public health, particularly for low-and middle-income countries deep in debt.

**Main text:**

D2H, as a practical health financing instrument, is not fully evidenced and gauged by academic literature though. We employed a five-step scoping review methodology. After posing questions, we conducted comprehensive literature searches across three databases and one official website to identify relevant studies.We also supplemented our research with expert interviews. Through this review and interviews, we were able to define the concept and structure of D2H, identify stakeholders, and assess its current shortcomings. Finally, we proposed relevant countermeasures and suggestions.

**Conclusion:**

This paper examines the D2H project's implementation structure and influencing variables, as well as the current research plan's limitations, with a focus on the role health funding institutions have played during the project's whole life. Simultaneously, it examines the interdependencies between debtor nations, creditor nations, and health financing establishments, establishing the groundwork for augmenting and revamping D2H within the ever-changing worldwide context of health development assistance.

## Background

Developing economies have been plagued by financial problems for many years, which has led to multiple societal crises [1]. The average debt stock of low- and middle-income countries (LMICs) increased by 5.6% from 2021 to 2022, reaching a total of US $9 trillion at the end of the year, according to the World Bank’s International Debt Report for 2022 [2]. According to the analysis, by 2022, the most poorest countries in the world will owe more than US $62 billion in external debt, a 35% increase from the 2021 figures [3]. Due to economic recovery, the overall debt service for LMICs in the post-COVID-19 era has decreased to 26% of their gross national income (GNI). However, rather than a decrease in the overall debt load, this shift is primarily due to the growth in GNI in 2021 [4]. Further evidence points to a potential crowding-out effect of high levels of public debt on health spending in LMICs [5]. The amount spent on health care worldwide in 2019 was $8.5 trillion. About 60% of this came from government funding, 40% from local private sources, and only 0.21% from outside help [6], suggesting that the primary external funding sources for sustainable development goals had reached a standstill. As a result, debtor nations' institutional and policy environments have gradually gotten worse [7, 8], and health financing is now much more constrained. Debt-to-Health (D2H) swaps, which have a history of successful transactions, show potential for addressing the twin issues of rising health expenditures and debt repayment in LMICs.

Clarifying the D2H creation and development process can be aided by the academic and grey literature now in existence.D2H refers to an agreement between debtor and creditor nations in which the creditor consents to give up some, or sometimes all, of the outstanding debt. The proceeds from these cuts are subsequently used to support medical programs and healthcare systems in the debtor nations. This program helps countries with limited resources by reducing their debt load and accelerating the mobilization of resources toward universal health coverage (UHC) [9, 10]. The Global Fund launched the D2H project in 2007 with a track record in debt conversation for health. Concerns regarding the fictitious nature of the project funds transfer, the replacement of intervention measures from other donors, the small scale of operations, arbitrary and disorganized planning and implementation, and the excessive specified uses initially cast doubt on the project's progress [11]. In the meantime, the Global Fund believed that the operation was complicated due to the low amount of cash mobilized by D2H and the high costs associated with transactions. As such, it was put on hold from 2011 until 2017 as a result of the need to keep looking for new financing sources as it encountered more and more fundraising obligations. After a hiatus for six years, it was resurrected in 2017 [12]. By the beginning of 2023, D2H programs had effectively converted some debt into additional health funds for LMICs. Moreover, D2H has enabled increased investments in strengthening health systems by providing debtor countries with access to additional fiscal resources.

Even though D2H’s efficacy has received widespread international recognition, we haven’t yet created a thorough review article to methodically arrange and assess it. This might be because there isn’t much accessible in the way of case studies and literature on D2H, and the focus is more on actual operations. Therefore, in order to support D2H's upgrading and ongoing development, it is imperative to construct a thorough operational and assessment framework. In order to determine the operational framework of D2H, a scoping review technique is used in this paper. The analysis centers on the functional role of health financing institutions as important players and their influence on the distribution of responsibilities between debtor and creditor countries.

## Main text

### Method

**Fig. 1:**
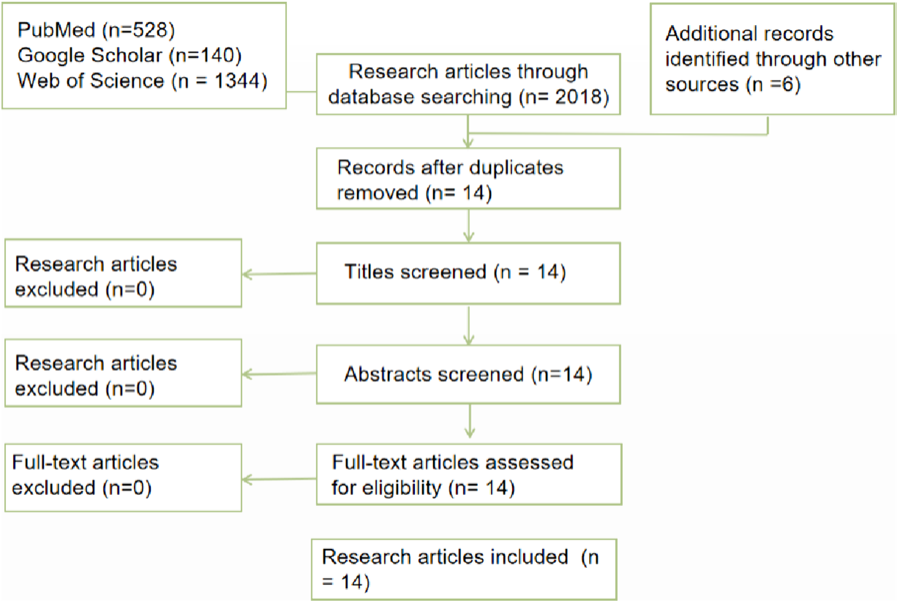
The flowchart of the research

This paper adopts the five-stage framework of Arksey and O'Malley as the research method of scope review [13], with the advantage of improving the credibility of the article when qualitative and quantitative evidence is insufficient [14], mainly including the following steps:

1. Clearly defined research questions: Understanding D2H’s workings and many advantages as a useful health finance instrument is the aim of this study, which also aims to offer a more comprehensive literature reference for next quantitative research.
2. Determining the research objectives: The reference literature and the web and policy materials comprise the two sections of this exploratory qualitative study. This is due to the fact that D2H is mostly utilized in practice, and there is currently little literature on the subject. The terms “Debt2Health,” “D2H,” and “debt-to-health swap” were combed through three of the largest databases: Web of Science, PubMed, and Google Scholar.We found six online resources and policy texts by searching websites, such as the Global Fund’s official website.
3. Literature search: Through retrieval, we included 2012 studies (140 from Google Scholar, 528 from PubMed, and 1344 from Web of Science).We also included 6 additional records identified through other sources.
4. Literature screening: By excluding content unrelated to the review, we included 17 studies. We excluded 3 duplicate studies and ultimately included 14 articles. The flowchart is as follows: (Fig. 1).

5. Literature Analysis: Through in-depth analysis of the selected literature, relevant information was extracted, categorized, and organized. It was found that there is currently a lack of comprehensive reviews on D2H. The purpose of this article is to fill the research gap on D2H.

This study also uses interviews as a supplement, interviewing the experts involved in the design of debt swaps, which provides a good complement to the design architecture, profit model, and existing shortcomings of D2H.

## Result

### D2H and its benefits

D2H originates from Debt-for-Development (D2D) swaps. It is noteworthy that in the post-World War II era, postcolonial governments in Latin America often generated foreign exchange by exporting ecological resources, such as rubber and timber, despite the region's simultaneous challenges of ecological catastrophes and debt. In 1984, American academic Thomas Lovejoy proposed a novel concept: a debt-for-nature swap [15]. According to data from national development finance estimates, the debt-for-nature method produced $126 million in local currency between 1987 and 1997 from around $134 million in commercial developing country debt, which was bought at an average discount of 78% [16, 17]. Pilot programs were started in the nations with large sovereign debt loads as a result of this effective strategy. The Economic Commission for Latin America and the Caribbean's endeavor to implement a debt swap that is climate adaptable since 2017 is one example of the situation in question. This plan uses a debt swap model similar to Belize's to lower the debt of three pilot countries by $527 million through the issuing of green bonds.

D2H is viewed as a subset of debt-for-sustainable development since it has proven to be effective in reorganizing debt and creating win–win scenarios for the creditor, the debtor nation, and a third party acting as an intermediate health finance organization. Since 2007, the Global Fund to Fight AIDS, Tuberculosis and Malaria (the Global Fund) has demonstrated the effectiveness of debt-to-health (D2H) swaps, and considerable progress has been made in raising more funds for health [18]. Debtor countries pledge to strengthen their health infrastructures or take up measures to fight AIDS, malaria, and tuberculosis, with the support from the Global Fund. These nations are released from their financial responsibilities in exchange from the creditor nations [19]. To date, the Global Fund has channeled investments over 226 million US dollars into debt conversion via D2H in the health sector for over ten countries, including Ethiopia, Egypt, Cameroon, and most notably Indonesia.

1. Generic operating procedure of D2H

**Fig. 2:**
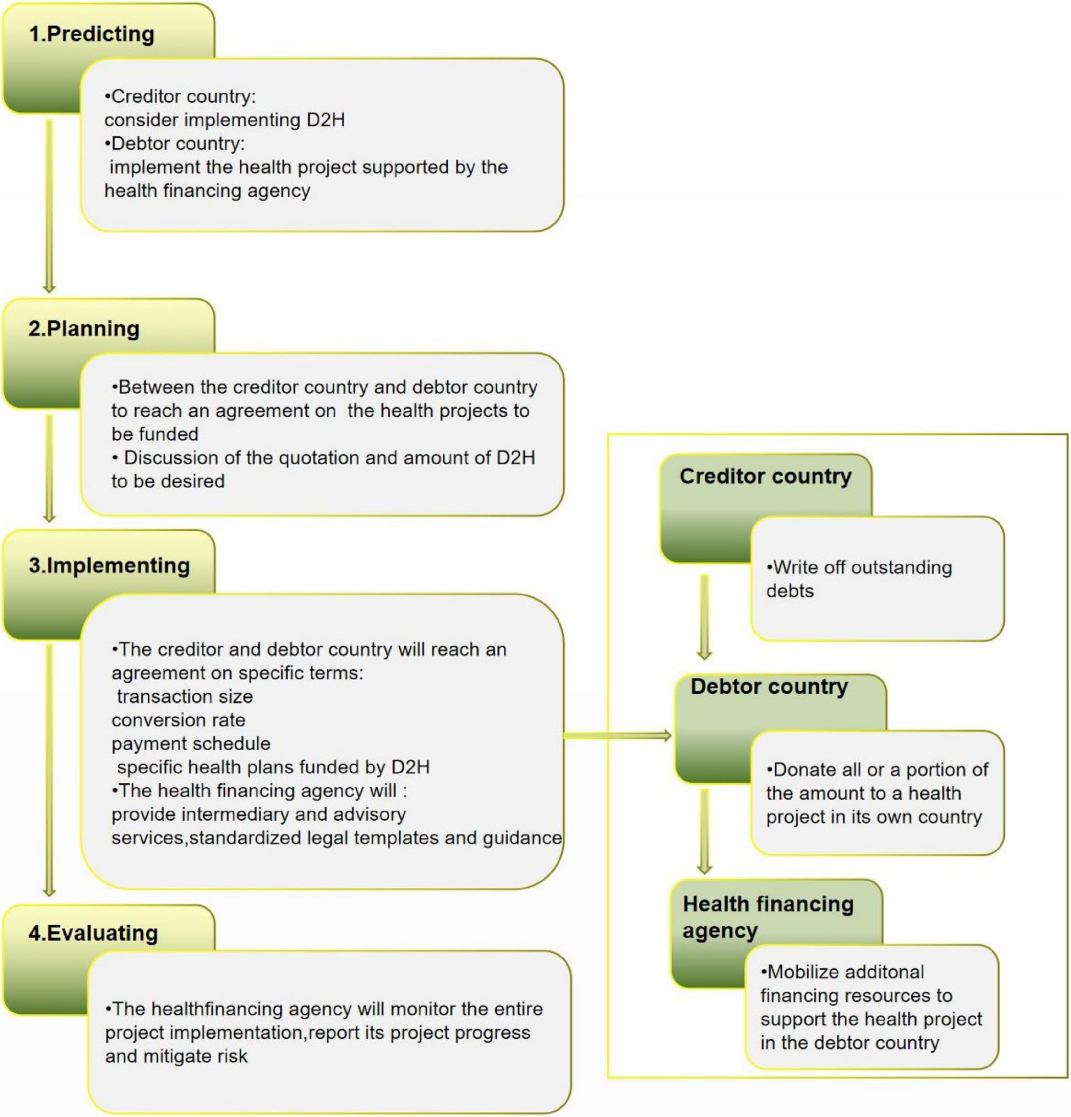
Generic operating procedure of D2H

A D2H scheme typically consists of four components (Fig. 2): anticipating or identifying potential opportunities for cooperation, planning, implementing, and evaluating. This involves reaching a partial or complete debt relief agreement between the debtor and creditor country through the mediation and coordination of a health financing agency, transferring the relief fund to the debtor country to support a particular health project, and the debtor country fulfilling its obligation to ensure that the relief fund is used appropriately to achieve the agreed-upon outcomes.

The intermediary health financing agency monitors the implementation of the health project, promotes transaction between the creditor and debtor country, and conducts administrative management and results reporting for both the creditor and debtor country.

**Fig. 3:**
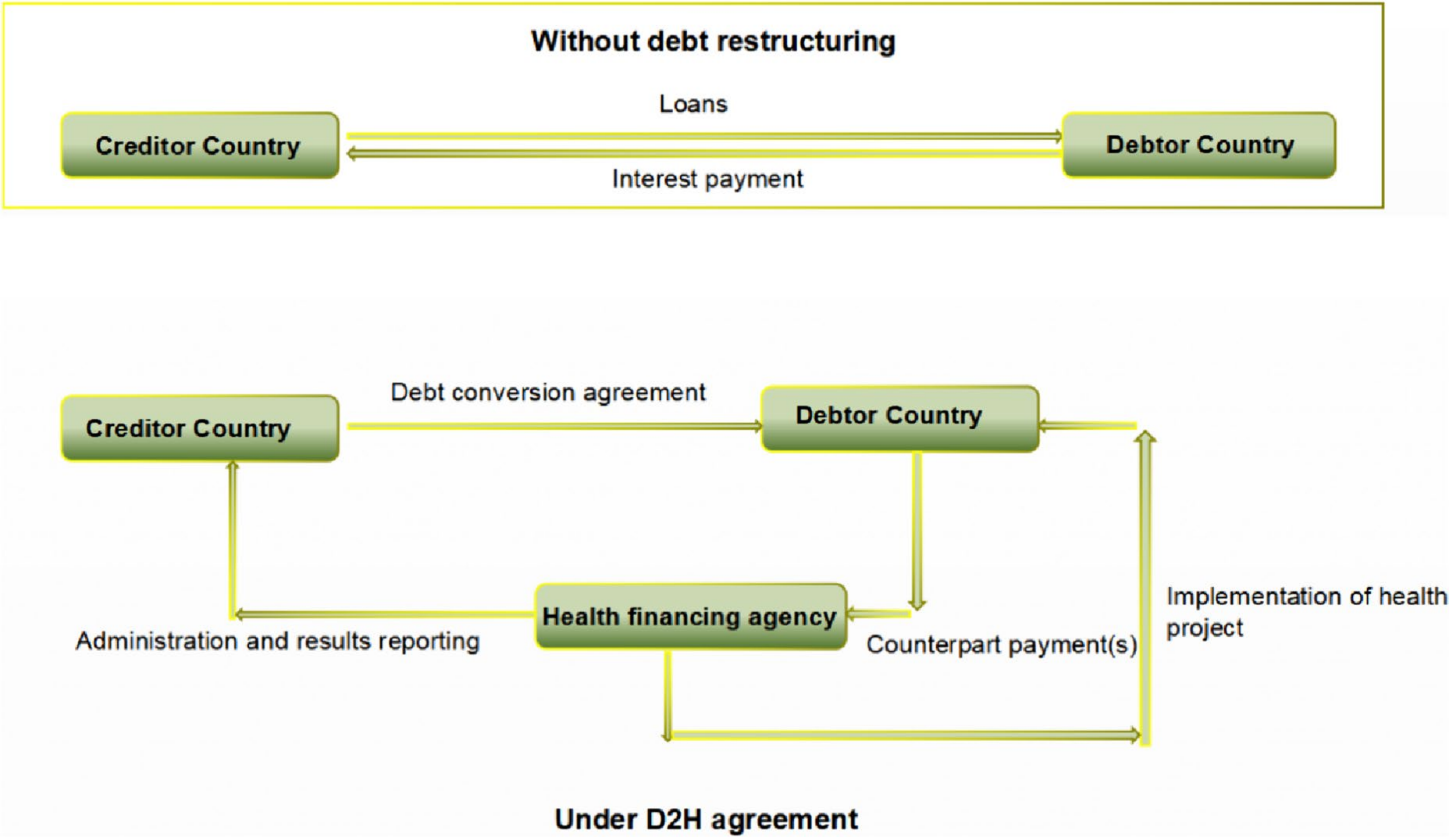
Comparison between the basic model of D2H and regular debt repayment

These institutions play a pivotal role as the "hub" of the D2H project. Taking the Global Fund as an example, first, the health financing agency serves as a “multilateral platform” that promotes health system strengthening, the fight against AIDS, tuberculosis, and malaria through multi-country donations and participation of over 100 countries, providing large-scale opportunities for D2H projects. Debtor countries and creditor countries can rely on this platform to negotiate and reach consensus. Secondly, the health financing agency functions as a “savings pool”. The negotiation and signing of agreements for D2H projects can be lengthy, but the Global Fund has a large number of health projects waiting for funding, ensuring that project implementation is not disconnected from capital investment. Finally, the health financing agency serves as a “monitor”. The implementation of D2H projects requires the exclusion of stakeholders. Although the Global Fund is stakeholder-related, its transparency and accountability as a humanitarian platform enable it to have a higher monitoring and evaluation effect, far superior to spontaneous monitoring and evaluation behaviors by debtor countries and creditor countries. The pattern diagram is shown in Fig. 3.

For instance, the Global Fund oversaw and carried out the Spain-Cameroon D2H project, which made it possible to clearly identify the tasks that each party played. Spain and Cameroon reached a debt reduction deal that resulted in the total cancellation of outstanding obligations of 24.1 million euros. About 40 percent of this total, or 9.3 million euros, came from Cameroon's donation to the Global Fund, which was then included in the nation's grant distribution. Through this 2017 agreement, the Global Fund's allocation for HIV and AIDS in Cameroon was increased to 81.7 million euros, enabling an additional 38,000 people living with HIV (PLHIV) to get antiretroviral medication.

**Fig. 4:**
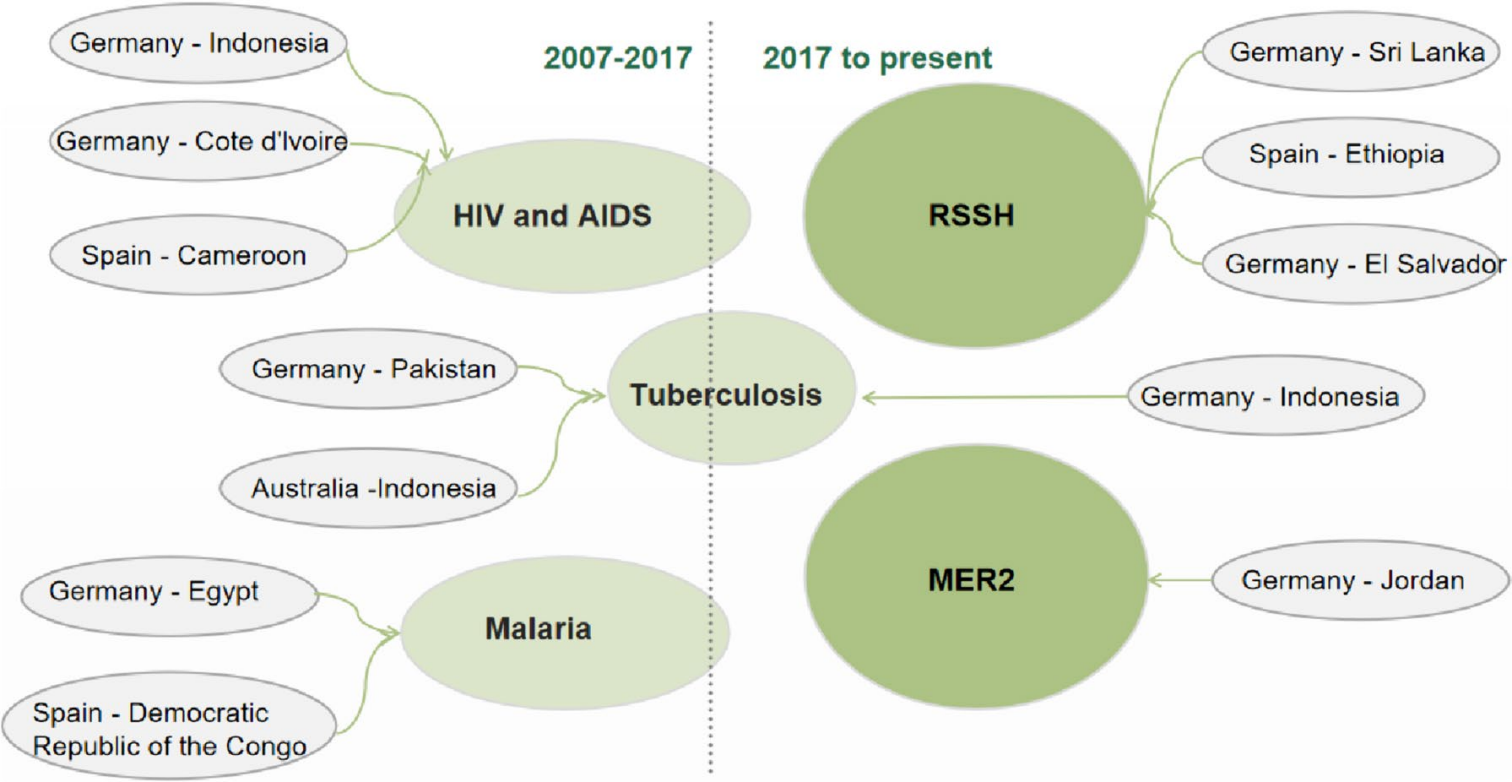
Evolution of D2H facilitated by the Global Fund(RSSH: resilient and sustainable systems for health MER2: The Global Fund’s Middle East Response2 program)

Increasing political resolve to tackle "horizontal" challenges like service delivery and health system strengthening is another important component of the D2H strategy. Eight projects supporting Indonesia, Pakistan, Egypt, Jordan, and Sri Lanka were part of the D2H exchanges between 2007 and 2021, with Germany acting as the creditor and the Global Fund acting as the intermediary. From the Global Fund's primary focus on major infectious diseases, the building of resilient and sustainable health systems (RSSH) was added to the scope of funding. Concurrently, the recipient program moved from discrete initiatives to a more comprehensive strategy, increasing its engagement in all-encompassing health initiatives [18] (Fig. 4). It should be noted that 2017 is not a turning point, for instance, the prevention and control of  tuberculosis continues to be the primary source of funding for D2H projects in Germany and Indonesia beyond 2017.

2. Benefits of D2H for different stakeholders

**Fig. 5:**
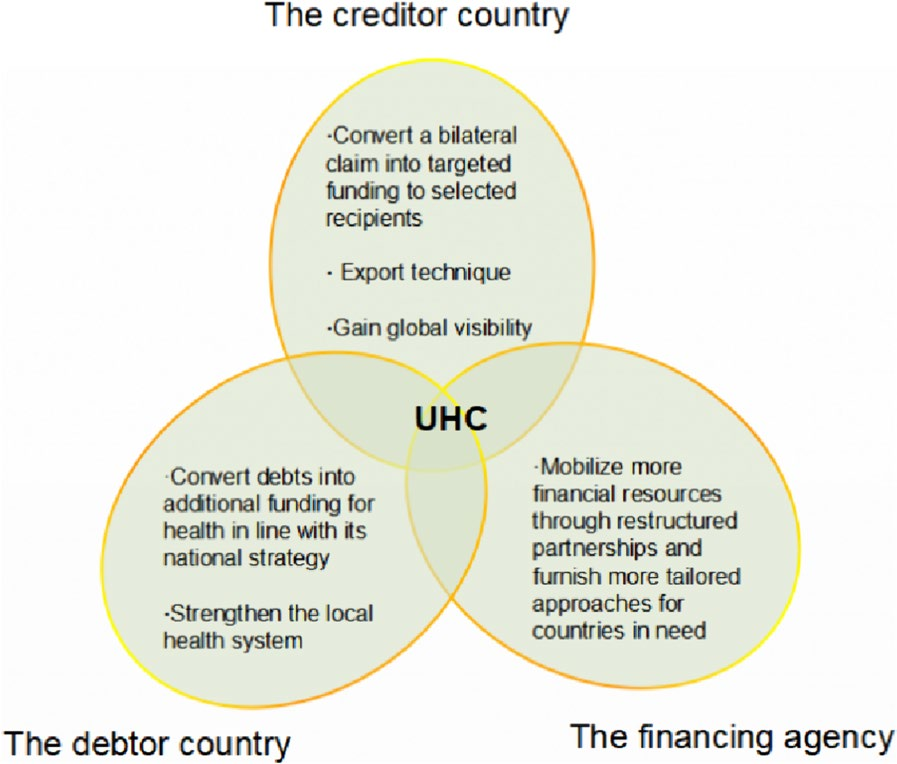
The added value of D2H

D2H, as a sovereign debt conversion solution, can create a triple win situation by adding value to all involved stakeholders (Fig. 5).

For the creditor country, D2H can serve as an opportunity to promote their own global public goods for health in the developing countries.Particularly considering the significant gaps in funding for health research, the growth of diseases affecting the poor, and the enhancement of epidemic prevention [20]. In particular, creditor nations may better export their human resources in the health sector, and initiatives like technology transfer will help creditor expertise go farther. In the meantime, the creditor nation raises its profile internationally and transforms a bilateral demand into targeted support for particular recipients. As an illustration, the D2H project was launched in 2019 between El Salvador and Germany. El Salvador’s $11 million debt was changed into $11 million for the building of the National Reference Laboratory (LNR) (https://www.theglobalfund.org/en/news/2022/2022-08-22-global-fund-approves-emergency-funding-to-maintain-essentialhiv-services-in-sri-lanka/) The lifecycle surveillance of the population, quick response to medical emergencies, and illness diagnosis and monitoring are all greatly aided by the LNR. Beyond just providing financial support, Germany is directly involved in building the LNR as part of this initiative. First, by improving ties with the National Institute of Health, Germany has reinforced technical guidance at the El Salvador LNR. Secondly, more diversified disease detection equipment, including those for rubella, dengue fever, and rabies, has been added in addition to improving conventional equipment for the detection of HIV, malaria, and tuberculosis. Lastly, because D2H has deftly converted sovereign debts into ODA investments, Germany, the largest D2H creditor country at this point, unquestionably maintains its leadership position in both the banking and health sectors [21]. This proves that creditor countries' gains in D2H initiatives—such as knowledge transfer, product exports, and improved international standing—are real and not just theoretical.

For the debtor country, it converts debts into additional funding for health in line with its national strategy. As seen in Fig. 3 above, there is a clear time breakpoint. Prior to 2017, the three main diseases of AIDS, malaria, and tuberculosis were the only ones receiving worldwide funding for D2H. But when the D2H project was halted and adjusted, the investment areas dramatically grew to include health system strengthening, with the goal of being in line with the nation's national health plan. The primary goal of the $16 million D2H project funds from Germany and Sri Lanka in 2021 was to reinforce the nation's quality management system and health information system, in addition to the primary health care strengthening initiatives being carried out by the Asian Development Bank and the World Bank. The areas that the RSSH, which is a macro concept discussed in this article, designates for assistance must be equitably negotiated by the three parties and be in line with the debtor nation's national health plan. Furthermore, D2H has advanced to the point where vertical and horizontal programs are integrated, which could support the local health system's strengthening. Using the D2H project between Germany and Sri Lanka in 2021 as an example, the Global Fund approved an emergency fund of US$989,687 in August 2022 to ensure the availability and distribution of necessary drugs and health care services for HIV treatment and prevention throughout Sri Lanka, in addition to strengthening primary health care projects (https://www.theglobalfund.org/en/news/2022/2022-08-22-global-fund-approves-emergency-funding-to-maintain-essentialhiv-servicesin-sri-lanka/). Later, to address financial shortfalls for the D2H project, an additional $4 million was supplied under the Global Fund's COVID-19 response mechanism. In order to better support the SDG targets of debtor countries, the D2H supported horizontal projects gradually complement its vertical projects, which focus on individual infectious diseases. 

For the intermediary health financing agency, it will be equipped with an effective instrument to mobilize more financial resources through restructured partnerships and furnish more tailored approaches for countries in need. For instance, increasing funding for initiatives against HIV, TB, malaria, and RSSH through the Global Fund helps save lives. The intermediary health financing agency serves as more of a "hub," as was previously mentioned. The amount of money given by D2H is insufficient to pay down a nation's health debt. It emphasizes gradual consequences more so than debt alleviation. D2H’s primary objective is to raise more funds for healthcare, and their influence on health takes precedence above debt reduction.

### Gap of the studies of D2H

1. Lack of data collection

As of now, the data we can collect from the Global Fund is as follows (Table 1). It is evident that only the countries of the debtors and creditors, the anticipated investment amount, and the actual amount received are now covered by the D2H data gathered by the Global Fund. We don’t have precise information about how money is used in the health sector. The vast range of D2H programs contributes to the paucity of information regarding their effects on health. Investments in laboratory building and health information systems, for example, produce various indicators related to health outcomes. Similarly, distinct health outcome metrics are produced by investments that target different diseases. There is presently no common system for measuring the many health initiatives that are carried out in different debt-ridden nations, despite the fact that performance evaluations have a major influence on the budget for the upcoming cycle of investments. Furthermore, the health policies of each debtor country influence how transparent D2H initiatives are. The current situation where data is predominantly provided by health financing entities like the Global Fund is the outcome of the intricate relationships among numerous stakeholders. Financial assessments are frequently given precedence over the assessment of protection outcomes in certain D2H programs that are protection-focused. Therefore, oversight by the health financing organization is still necessary to address the problems of data scarcity and lack of openness. Also, under the direction of the health financing organization, debtor and creditor countries should engage in developing precise, open, and mutually agreeable indicator systems and offer concrete data.

**Table 1:** Percentage of D2H in the contribution by creditor countries to the Global Fund from 2008–2019 (Source: the Global Fund database Currency:USD)

Creditor country	Period	Contribution	Percentage
Australia	2008–2010	1,849,875	0.052
	2011–2013	12,332,754	0.350
	2014–2016	21,084,861	0.598
	2017–2019	0	0
	**Total**	**35,267,490**	**1**
Germany	2008–2010	35,266,606	0.209
	2011–2013	30,787,051	0.183
	2014–2016	6,441,883	0.038
	2017–2019	95,883,010	0.569
	**Total**	**168,378,550**	**1**
Spain	2008–2010	0	0
	2011–2013	0	0
	2014–2016	0	0
	2017–2019	17,441,608	1
	**Total**	**17,441,608**	**1**

2. Lack of summary of lessons learned and adequate evaluation of the project mechanism and impact

Both qualitative and quantitative methodologies must be used in tandem to create a thorough evaluation system for the D2H project. A thorough examination of the project's effective components and underlying logic can be achieved through qualitative evaluation, which can also yield valuable insights for project optimization. A more objective and accurate representation of the project's operational effectiveness can be achieved through quantitative evaluation, which is grounded on data and offers a more scientific foundation for decision-making.

Qualitatively, there isn’t a summary of the D2H project's past successes as an endeavor. Conducting a methodical survey of designers, implementers, and evaluators is necessary for this. The D2H mechanism can be further optimized and improved by following the overview of successful D2H experiences. Quantitatively, a data-driven evaluation plan is necessary for the whole D2H project phase. There are four stages for the quantitative evaluation. The first step, known as the feasibility review, calls for debtor and creditor countries as well as the health financing organization to evaluate the available funds, how they will be used, and when they will be disbursed. The second phase, known as process assessment, evaluates the elements that drive project execution, such as resource allocation, policy environment, and implementation efficiency. Impact evaluation, the third step, evaluates the project’s actual operational effectiveness in terms of illness control, health improvement, and economic impact. The project's long-term stability and sustainability are primarily evaluated in the fourth stage, sustainability evaluation, which also determines whether more funding or policy assistance is required.

### Typical features and factor for a successful D2H

Through scoping review and expert interview, we can summarize some typical features of D2H:

1. A strong political will

The most important element influencing D2H’s effectiveness is the political will of both the creditor and debtor nations. The following factors are typically seen to be essential when choosing a debt reduction option:1). The global political debt relief decisions may be hampered by strained international relations, which could erode the political will of both debtor and creditor countries [22] 2). Debtor countries’ economic circumstances: Making decisions also requires consideration of the debtor countries’ economic circumstances. Political authorities may be more receptive to debt relief if the debtor country's economy is in decline and it has little budgetary room for SDG-related expenditures. 3). The creditor countries’ readiness: It is reasonable to expect creditor countries to support debt reduction, and this is one of the deciding criteria as well [23]. 4). The Global Fund and other health financing organizations play a crucial leadership and coordination role in advancing D2H by bringing together the governments of the debtor and creditor countries to facilitate negotiations and ultimately lead to a mutual agreement. In other words, sincere respect for and consideration of the political goals of all participating nations are necessary for making programs successful. D2H projects also need to be assessed from a number of angles, including political dynamics, credit ratings, the debtor nation's foreign debt policies, its capacity for implementation, and the supervision and coordination of the third party during the entire undertaking.

2. Debt transparency as an essential prerequisite

The international community is acutely aware of the necessity of debt transparency, as demonstrated by the “Tuna Bond” episode in Mozambique, which underlines the dangers of inadequate debt transparency [24]. In order for D2H schemes to be configured, debt transparency is required. This helps evaluate whether debt relief is required by giving all involved parties a clear image of the outstanding debts, risks of debt default, and the status of fiscal incomes and expenditures. Without openness, donor nations may find it difficult to formulate a sensible plan to assist recipient nations in reducing their debt load, which could lead to agreements that are untrustworthy or deceptive. Debt transparency has been shown to improve how money is allocated and used, protect against financial errors, and stop resources from being abused or wasted. Transparency in debt can guarantee that D2H financing is directed toward high-priority areas through well-managed and supervised projects. Moreover, debt transparency will boost investors' confidence in making long-term investments by improving their capacity to recognize particular projects and manage risks.

3. Administrative changes pose a burden on both parties

The complete integration of financial, material, and human resources is necessary for the successful deployment of D2H. These could include: 1). The debtor country must set aside money for D2H in order to ensure prudent fund management and focused investments. 2). The debtor country should conduct initial evaluations to determine the project's feasibility and dangers, as well as to describe the investment plan and expected financial gains. 3). Establishing a strong oversight system, keeping a close eye on the project's development, and quickly identifying post-project risks and issues are critical. Timely improvement of project management is just as vital as regular, evidence-based audits and evaluations of the project. 4). Every involved party should devise a thorough system of investor responsibility while providing them with necessary services such as training courses. 5). To guarantee a seamless implementation, the debtor nation must raise understanding of D2H-related policies, operational procedures, and its impact.

### Uncertainties and risks associated with D2H

1. Lack of willingness and determination of stakeholders

D2H can be viewed as a subdomain of debt swap. Creditor nations exhibit varying perspectives regarding debt swap.Using Germany and France as examples, Table 1 of this article and the Global Fund's database both attest to Germany's long-standing involvement in the D2H project in the health sector and its active participation in the debt exchange mechanism. In order to support development in fields like education, health, infrastructure, and the preservation of natural resources, heavily indebted impoverished nations can lower their debt through France's “Debt Reduction for Development Contract (C2D)” [25]. The fact that this framework already exists and is similar to the debt swap mechanism may be the cause of France's exclusion from the D2H project in the health sector.

Success depends on a number of contributing factors, including as the monetary policies of debtor countries, economic growth, and political stability. Furthermore, it is important to keep in mind that the drawn-out process of designing, implementing, and achieving a return on investment for a particular D2H project may weaken the resolve of potential partners.

2. Concerns about credit rating and refinancing ability

Due to the debt cancellation, D2H could result in the debtor country's credit rating being lowered. Everyone is aware of the importance of a nation's sovereign credit in determining the nature of the world's financial and economic systems. This credit rating reflects the country's position within the global financial system. A country's ability to borrow money internationally is strongly influenced by the level of its sovereign credit rating [26]. Modifications to this rating may have an effect on foreign financial liquidity and, consequently, the stability and security of the world's financial markets. Large sovereign rating companies like Fitch use a multiple regression model for scoring, whereas Moody's and S&P often use a scoring table and matrix. The assessment metrics encompass domains such as financial stability, political clout, economic potency, and exogenous hazards. Like the Debt Service Suspension Initiative (DSSI), several developing countries are hesitant to participate in the D2H framework because they are concerned about both the costs and possible effects on their sovereign credit ratings.

3. On the delicate balance between creditor countries' power and debtor countries’ sovereignty

With Germany as an example, the D2H initiative illustrates Germany's distinct approach to managing debt ties with developing nations. It serves as a model for Germany's health assistance. It is nevertheless worthwhile to investigate further to see if such a process design is indeed fair and takes into account the debtor countries' actual demands and decision-making authority.The Federal Ministry for Economic Cooperation and Development is responsible for the qualifying examination and initial design of debtor countries in debt swap arrangements (BMZ). Debtor countries are negatively impacted by being “selected” to some extent because of the process's obvious subjectivity and selectivity. However, this does not imply that debtor countries are voiceless during the process. In actuality, the health issues that creditor countries seek to address are typically the outcome of fair triangular consultations and are in line with the national health policies of debtor countries. By creating a win–win situation, these agreements guarantee that aid money are actually used to raise the health standards of debtor countries. Institutions that provide third-party finance for health care, like the Global Fund, are essential to this process. In addition to being in charge of carrying out aid programs precisely, they also have to turn in yearly reports or reports from outside audits to guarantee compliance and openness with the use of money. These organizations safeguard the sovereignty of debtor countries by somewhat balancing the authority of creditor countries. They guarantee the efficient utilization of aid monies and avert any corruption and embezzlement by closely monitoring and assessing programs. Overall, reasonable consultations and the balancing role of third-party health financing institutions enable a relatively equal cooperative relationship between creditor and debtor countries, even though the D2H project may disadvantage debtor countries in terms of process design. Nonetheless, given the dynamic nature and the growing intricacy of international relations, the question of how to enhance this model and guarantee its impartiality, efficacy, and durability continues to be one that merits consideration and investigation.

### Discussion of how to catch up on the research into D2H

**Fig. 6:**
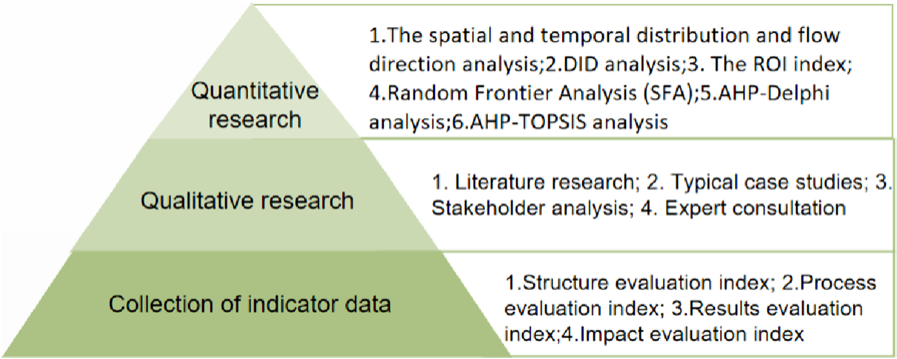
Catch-up on the research into D2H

Lack of systematic research into D2H may hinder the progress of D2H in practice. We propose the following analytic methods to be applied for an in-depth study of D2H: (Fig. 6).

1. Collection of indicator data

For both the qualitative and quantitative evaluation of D2H schemes, the evaluation criteria should be set, which involves detailed data. We need well-structured evaluation index (population, economy, social environment, project schedule, budget, etc.), process evaluation index (D2H-generated fund distribution, specific implementation process, etc.), results evaluation index (death, composite health evaluation index, health service utilization index, etc.), impact evaluation index (Gini coefficient, healthcare access and quality index, international health regulation index, infant survival rate [27], cost-effectiveness analysis, etc.). It is worth noting that data collected varies between different D2H schemes.

2. Qualitative research

Literature research method is crucial for the development of D2H analysis. At present, the research results on D2H financing mode, operation mechanism, applicable scenarios and conditions, key stakeholders, effects and effects are all based on content analysis, expert consultation, swot analysis and bibliometric analysis. Qualitative research can help to clarify problems by drawing on the hands-on experience and feedback from different stakeholders and lay the ground for the subsequent quantitative research.

3. Quantitative research

In general, D2H is a policy-based approach involving various parties. The scale of funding, measurable SOPs, and impact are the focal areas of quantitative analysis.

The traditional analytical methods are described as follows: As for funds, the spatial and temporal distribution and flow direction analysis can be used on the basis of detailed data [28]. Regarding the D2H item effect, we can perform the DID analysis through the time breakpoints.

Non-traditional analysis can be as follows: In the face of the economic attributes of the decision on D2H, the ROI index can be used to evaluate benefits, and make the next budget planning and strategy formulation [29]. Random Frontier Analysis (SFA) is used to estimate the technical efficiency score of health systems. We can specify a regression model to evaluate the effectiveness of creditor countries' investment in technology efficiency [30]. But in order to study D2H more thoroughly, combined qutlitative evaluation methods need to be applied. For example, AHP-Delphi analysis is used to screen the indicators, determine the index system and give weight. AHP-TOPSIS analysis method is to determine the fit of the evaluation object between the positive and negative ideal solutions to determine the optimal evaluation between multiple experts [31, 32].

More importantly, it is desirable to screen out the outcome indicators that can well evaluate the impact of D2H, which is of great benefit to the econometric analysis of the influencing factors of the panel data and contributes to the development of future schemes. Of course, attention should be paid to the fact that the outcome evaluation indicators of different projects vary greatly and will be customized.

## Conclusions

In this study, we lay forth a precise definition of D2H, create an operational and assessment framework, and highlight its importance in the field of health funding. The role of a "multilateral platform" makes them a viable option for large-scale D2H projects, facilitating negotiations and agreement-making between debtor and creditor countries. Health finance organizations will make ensure that project execution and capital investment are linked as a "savings pool." It is more effective to have "monitors" in monitoring and evaluating projects than what the debtor and creditor countries would do on their own due to their responsibilities. As a "balancer" between debtor and creditor countries, debtor countries can avoid being in a passive position and have the freedom to select aid projects that complement their national health policies, even though creditor countries have priority in choosing debtor countries and investment amounts. This is made possible by the oversight and checks and balances provided by health financing institutions. We do, however, also highlight the difficulties the D2H project is now facing. The demand for health finance is rising while the supply of funds is getting tighter due to the shifting global health landscape and the unequal economic development of nations. The methodical modernization of the D2H initiative for global health will depend heavily on our suggested operational and assessment frameworks. The project team will be able to better manage the project's development by using the operational framework to determine the necessary processes and deadlines for each task. The program will be regularly monitored and evaluated thanks to the evaluation framework, allowing for the assurance that everything is operating as planned and that the strategy is being adjusted to the ever-changing environment. To jointly further advancement in the area of global health funding, the D2H initiative must in the future improve communication and collaboration with partners including governments, non-governmental organizations, and international organizations. Through mutual understanding, information exchange, and resource management, we will collaborate to overcome obstacles and accomplish shared objectives.

## Figures and Tables

**Fig. 7:**
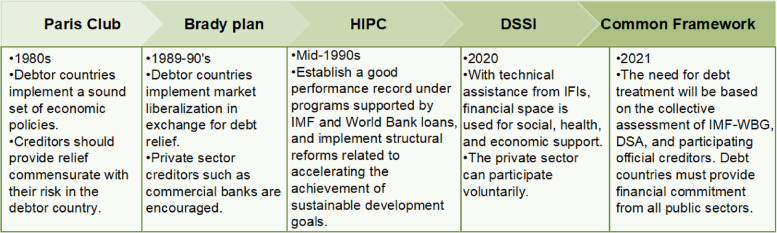
Global actions to address sovereign debt issues since the 1980s (HIPC: heavily indebted poor countries initiative DSSI: Initiative to suspend debt repayment)

**Fig. 8:**
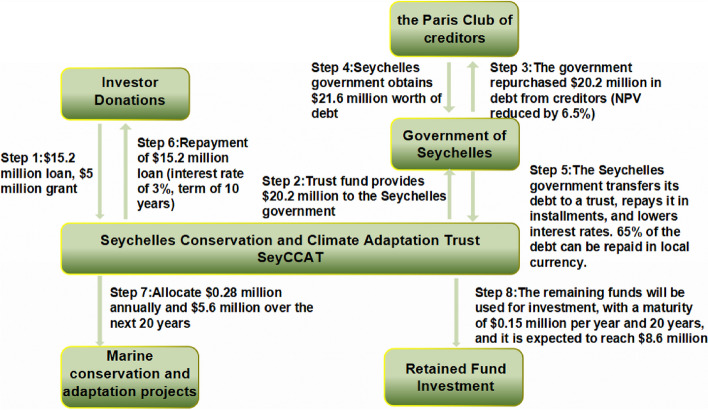
Seychelles debt for nature project flowchart

## Data Availability

All data sources are referenced and in the public domain.

## Appendix 1

In response to sovereign debt default in LMICs, there is no formalized bankruptcy framework at the global level. Instead, the international community predominantly relies on contractual mechanisms to solve such crises [33]. As shown in Fig. 7, since the inception of the Paris Club in the 1980s, there have been concerted global efforts to mitigate sovereign debt burdens [34]. These initiatives endeavor to harmonize the interests of both the least developed nations and their creditor counterparts. The primary objectives are twofold: to ensure the sustenance and developmental rights of the least developed countries and to fortify the overall stability of the global financial system.

## Appendix 2

To further develop D2H, we may learn from the Seychelles environmental protection project, as shown in Fig. 8. From the perspective of Seychelles project, the capital volume of debt-for-nature project is small, and the original intention of the design is not to solve all debt problems, but it can leverage debt conversion and obtain reputation premium. With the subsequent impact of the Seychelles project as an example, apart from the protection benefits of the marine project, it also activated the tourism and fishery [35].

The debt-for-nature project in Seychelles is an innovative debt conversion approach, with its innovation mainly reflected in the following aspects [36]: 1). Combining multiple nature conservation measures: The debt-for-nature project in Seychelles is not a single nature conservation scheme, but rather a combination of various nature conservation measures. For example, it includes the construction and management of marine protected areas, the protection and restoration of terrestrial forests, and the promotion of climate change adaptation measures. 2). Highly targeted and sustainable: The debt-for-nature scheme in Seychelles is formulated based on the natural characteristics and development needs of Seychelles itself, thus possessing strong targeted characteristics. In addition, the project emphasizes the coordinated development of natural conservation and economic development, emphasizing the sustainability and economic benefits of conservation measures. 3). Multi-party cooperation and diversified financing: It is implemented through multilateral collaboration, including the government of Seychelles, multilateral development banks, international environmental organizations, and the private sector. The financing methods are also quite diverse, including government funds, international donations, and private investment in addition to debt conversion.

## Abbreviations

D2D: Debt-for-Development
D2H: Debt-to-Health
DSSI: Debt Service Suspension Initiative
GNI: Gross National Income
LMICs: Low-and middle-income countries
MER2: The Global Fund’s Middle East Response2 program
PLHIV: People living with HIV
RSSH: Resilient and sustainable systems for health
SFA: Stochastic Frontier Analysis
UHC: Universal health coverage
